# Correction of In-Patient Severe Hypernatremia in an 81-Year-Old Female With Hypopituitarism

**DOI:** 10.7759/cureus.51474

**Published:** 2024-01-01

**Authors:** Luke Henwood, Austin Vaughn, Ravish Narvel, Rahil Gour

**Affiliations:** 1 Medicine-OMS3, Lake Erie College of Osteopathic Medicine, Bradenton, USA; 2 Internal Medicine, Ascension St. Vincent's - Riverside, Jacksonville, USA; 3 Family Medicine, Ascension St. Vincent's - Riverside, Jacksonville, USA

**Keywords:** ddavp, acute metabolic encephalopathy, anti-diuretic hormone, co-morbid conditions, elderly population, osmotic balances, community aquired pneumonia, central diabetes insipidus (cdi), hypopituitarism, hospital acquired hypernatremia

## Abstract

Hypernatremia has been significantly associated with in-hospital mortality and discharge to long-term care facilities. The appropriate correction of electrolyte disturbances, especially sodium, is important to consider to prevent the addition of central nervous system disturbances, such as cerebral edema and eventual brain injury. The importance of maintaining a proper correction of hypernatremia has been well studied and used in clinical practice. Choosing to use a hypotonic solution is a key principle. It is of utmost importance to adjust the rate of correction based on the patient's symptoms, underlying etiology, and associated comorbidities. This case demonstrates how a correction formula was used and adjusted accordingly in an 81-year-old female with severe hypernatremia and metabolic encephalopathy with multiple comorbidities, including hypopituitarism. It is noteworthy to examine the correction rate, how it was calculated and delivered, and how the main cause of the hypernatremia was determined. Considering all these factors can help to properly administer any additional corrective medications, such as desmopressin (DDAVP) in a patient with diabetes insipidus (DI) secondary to hypopituitarism, or adjust the correcting rate based on signs, symptoms, and laboratory findings.

## Introduction

Hypernatremia, defined as a serum sodium concentration exceeding 145 mmol/L, represents an electrolyte disturbance that can seriously affect overall health and well-being [1,2]. It arises from an imbalance between water intake and loss, disrupting the body's water and sodium homeostasis. Hypernatremia presents with osmotic imbalances with clinical features that are caused secondarily by water shifting out of all body cells, including brain cells, leaving the patient dehydrated [3]. It is important to note that excessive water loss rarely causes hypernatremia because of the body's thirst response, which leads to hormonal interaction and increased water consumption, as discussed later [4]. As plasma sodium is a solute that cannot freely pass through cell membranes, it plays a role in the tonicity of the extracellular fluid, leading to the movement of water across cell membranes. Consequently, hypernatremia results in hypertonicity and invariably leads to temporary cellular dehydration [5]. The clinical signs and symptoms of hypernatremia primarily involve disruptions in the central nervous system due to the shrinkage of brain cells. These manifestations become more pronounced when there is a rapid or significant increase in serum sodium concentration. The range of hypernatremia symptoms can vary from thirst, weakness, increased neuromuscular excitability, hyperreflexia, lethargy, confusion, seizures, or coma. In cases of acute hypernatremia occurring within 48 hours, the sudden shrinkage of brain cells can lead to potentially severe consequences such as vascular rupture, cerebral bleeding, subarachnoid hemorrhage, or even fatality [5,6].

The body's complex regulatory mechanisms ensure the balance of sodium and water levels, primarily through urine concentration facilitated by antidiuretic hormone (ADH) and a robust thirst response promoting increased fluid intake. ADH binds to three receptors, V1, V2, and V3, all of which are coupled to G-proteins for intracellular modulation. The V1 receptor is mainly located on vascular smooth muscle and is essential for maintaining appropriate vasoconstriction. The V3 receptors are mainly located in the central nervous system, particularly in the adenohypophysis, regulating the release of corticotropin. Finally, and most importantly for electrolyte and water balance, the V2 receptor, which is located on the apical membrane of the kidneys, is responsible for aquaporin upregulation and subsequent water reabsorption [7]. Specific susceptible populations may experience impaired mechanisms, such as ADH deficiency or renal tubular unresponsiveness, compromising the body's ability to prevent hypernatremia [8]. Free water loss leading to hypernatremia can be seen in patients with central or nephrogenic diabetes insipidus (DI). In fact, according to Sonani et al., the main cause of hypernatremia is indeed total body water loss and hypovolemia, which can occur without a proper thirst response or ADH signaling [1]. Central DI occurs due to inadequate production of ADH. Some common causes of central DI are idiopathic, head trauma, cranial neoplasm, and pituitary infiltrative diseases, such as sarcoidosis and histiocytosis. The preferred therapy for central DI is desmopressin (DDAVP), an ADH analog. Treatment is usually maintained to prevent hypernatremia, depending on the cause of central DI [1].

As stated above, central diabetes insipidus can result from hypopituitarism, which can be defined as the partial or complete failure of the anterior and posterior pituitary hormones to avidly secrete their respective hormones, the most important of which for maintenance of volume and subsequently sodium is ADH [9]. In a patient who has partial DI, achieving an euvolemic state may be successfully achieved with reflexive thirst mechanisms and increasing fluid intake. A proper thirst mechanism needs to be considered if a decision to place the patient on DDAVP is made. In fact, according to Alexandraki and Grossman in 2019, if the patient has a proper thirst response, 25% of patients with partial central DI will develop hyponatremia due to the inability to reverse the antidiuretic effect of DDAVP when the person's fluid intake goes above their body’s requirements [9]. Thus, it is important to consider and understand the interaction between the hormone ADH, whether in its natural form or its synthetic form, its influence on volumetric homeostasis, and its ability to alter sodium levels in the body.

When a patient with an abundance of medical issues presents to the emergency department, the main focus is to address the most critical issue first, regardless of whether this is the patient's main concern. Electrolyte disturbances, particularly sodium, can vary largely in an acutely ill patient with multiple underlying comorbidities. What is of utmost importance is being able to understand how to properly correct the electrolyte disturbance, preventing further deterioration in the patient. A critical review of the patient's past medical history to determine why the electrolyte disturbance is occurring is the first step in the management of the patient. Next, it is important to consider other factors that play into the balance of sodium, such as ADH, aldosterone, glycemic hemodilution, kidney function, and so on. Finally, working in a step-like fashion to properly address the problem list is crucial to treating an acutely ill patient. If a patient presents with community-acquired pneumonia (CAP)-like symptoms, this should be treated first if it is the most critical issue at hand that can acutely deteriorate the patient. Community-acquired pneumonia can be a rapidly fatal condition, especially in elderly patients with comorbidities. Mortality has been found to be more than double in an elderly patient as compared to a younger patient [10]. In acutely ill patients, especially those with hypopituitarism, electrolyte disturbances are common. A decision to maintain a higher serum sodium level rather than a lower serum sodium level can be beneficial for most patients. Studies have shown that hyponatremia has been associated with a greater risk of respiratory failure due to osmotic imbalances, mostly from fluid overload with resultant pulmonary edema and hypercapnia [11,12]. On the flip side, hypernatremia has been shown to produce a worse outcome for patients with community-acquired pneumonia, causing seizures, coma, and eventually death with sudden shrinkage of cells from the osmotic pull of sodium [11,13]. Regardless, the decision is never really influenced by which direction of sodium one wants to go; instead, it is to monitor and understand how to correct the sodium to a normal range after the initial insult. Therefore, understanding the calculation for determining the water deficit to give a hypotonic solution to correct a hypernatremic, CAP, or DI patient is critical to preventing cerebral edema and coma. The equation is as follows: 

Total body water [0.6 in men and 0.5 in women × body weight (kg)] × [(plasma sodium/140) − 1]

and must be used with great precision to prevent an over-correction of sodium in a hypernatremic state, remaining at no more than a rate of correction of 12 mEq/L/day (< 8 mEq/L in the first 24 hours) [1,3]. In the event of rapid-onset acute hypernatremia, there is clinical evidence that using pure water orally, 0.45% sodium chloride intravenously, or 5% glucose intravenously allows for the safe correction of hypernatremia [4]. However, this may not fix the underlying etiology based on the cause of free water loss. Measures should be taken to ensure the possible underlying causes of hypernatremia are addressed.

## Case presentation

An 81-year-old Caucasian female with a past medical history of idiopathic panhypopituitarism, diabetes insipidus, and recurrent right upper lobe pneumonia in January and April presented to the emergency department with complaints of fatigue, generalized weakness, and shortness of breath. She appeared drowsy but was oriented to the person, place, and time upon assessment. She denied any past medical history of lung disease other than COVID in the past. She previously saw a pulmonologist and did pulmonary function tests, with which she was told her "lung functions are great." However, no previous PFTs were noted on record. The patient denied being on home oxygen or inhalers. She was admitted multiple times in the last few months and discharged with treatments for pneumonia. She reported being able to walk at home without shortness of breath. Per documentation, one of the presenting complaints was shortness of breath, so it was unclear if the patient was able to ambulate and/or was short of breath at home. A review of the systems was negative for any additional issues outside of the scope of treatment for underlying conditions. On the physical exam, she was lethargic but arousable. She had shallow, unlabored, decreased breath sounds on 2 liters (L) of oxygen administered via nasal cannula. She had minor musculoskeletal weakness, normal deep tendon reflexes, and cranial nerves II-XII were grossly intact.

A plan to draw labs and work up CAP was decided upon. She was given Rocephin intravenously (IV), which eventually was broadened to Cefepime following a pulmonary consultation. She was found to have leukocytosis and her at-home steroid medication was slowly discontinued to track the leukocytosis and response to antibiotics. Due to her chest radiograph showing mild heart enlargement as well as her presenting symptoms, she was started on Lasix 20 mg IV daily. 

After medication reconciliation, it was decided per nephrology to taper off her DDAVP that she was taking at home slowly due to heart enlargement secondary to suspected potential heart failure and fluid overload. In the ensuing days, her sodium levels began to slowly elevate. Her normal saline infusion was decreased from 125 mL/hr to 75 mL/hr and a stat complete metabolic panel was ordered, resulting in persistently elevated sodium. Her normal saline of 75 mL/hr was stopped and she was immediately placed on dextrose 5% in water (D5W) at 75 mL/hr. In addition, she was ordered to resume her hydrocortisone 20 mg every morning and 10 mg every night, as well as her levothyroxine 50 mg per day. Her IV Lasix was changed to oral Lasix at the same dose of 20mg. Over the next day, she developed supraventricular tachycardia with little urine output and was emergently treated with a 500-mL normal saline bolus. This resulted in no response and was followed by an additional 500-mL normal saline bolus with little response. An additional 1 L normal saline bolus was given, which finally slowed the heart rate. Her blood cultures returned and she was ordered to stop Cefepime and start Levofloxacin 750 mg for seven days. Her hypernatremia continued to worsen at 160 mmol/L and her D5W dose was increased to 100 mL/hr. She resumed DDAVP, and nephrology was consulted.

She then became very tremulous, with thick mucus coming from her mouth and a sodium level of 171 mmol/L. Her nasogastric (NG) tube was pulled out from 75 cm to 55 cm and she had a stat chest radiograph and KUB for aspiration and NG tube placement. She was given 200 cc/hr of D5W, which dropped her sodium from 171 mmol/L to 160 mmol/L in six hours, eventually reaching 156 mmol/L in nine total hours. For several hours, D5W was halted to allow for equilibration. Repeat sodium remained at 156 mmol/L and D5W was restarted at 50 cc/hr. There was no evidence of seizure activity. She was to remain at 50 cc/hr until her sodium dropped to 140 mmol/L and her sodium was monitored closely. Within three total days after a sodium spike to 171 mmol/L, her sodium dropped to 148 mmol/L. She was discharged in no acute distress and back to baseline with a sodium total of 145 mmol/L just four days after the increasing spike (Tables 1-4).

**Table 1:** Initial lab values on admission

Lab	Result
CBC	
White blood cell count	22 k/mcL high
Red blood cell count	3.28 million/mcL
Hemoglobin	11.2 g/dL low
Hematocrit	35.5% low
MCV	81.1 fL
MCH	25.7 pg
MCHC	31.7 g/dL
RDW - red cell distribution width	18.1% high
Platelet count	433 k/mcL
MPV	7.7 fL low
Neutrophils %	74 % high
Lymphocytes %	19 % low
Monocytes %	6 %
Eosinophils %	1 %
CMP	
Sodium level	137 mmol/L
Potassium level	3.2 mmol/L Low
Chloride	102 mmol/L
Carbon dioxide	24 mmol/L
Glucose level	97 mg/dL
Blood urea nitrogen	19 mg/dL
Creatinine	1.3 mg/dL
Anion gap	11 mmol/L
Osmolality calculated	288 mOsm/kg
Bilirubin total	0.6 mg/dL
Protein	7 g/dL
Albumin level	4.2 g/dL
A/G ratio	1.5
Calcium level	9.7 mg/dL

**Table 2:** Lab values four days after admission

Lab	Result
CBC	
White blood cell count	10.8 k/mcL
Hemoglobin	10.6 g/dL low
Hematocrit	35.8 % low
Platelet count	448 k/mcL
Basic metabolic panel with magnesium and phosphorous	
Sodium level	171 mmol/L critical
Potassium level	3 mmol/L critical
Chloride	141 mmol/L high
Carbon dioxide	19 mmol/L low
Glucose level	178 mg/dL high
Blood urea nitrogen	8 mg/dL
Creatinine	1.3 mg/dL
Calcium level	10.7 mg/dL high
Magnesium level	2.2 mg/dL
Phosphorus level	1.1 mg/dL low

**Table 3:** Lab values after correction with D5W

Lab	Result
CBC	
White blood cell count	14.2 k/mcL high
Hemoglobin	8.2 q/dL low
Hematocrit	27.1 % low
Platelet count	429 k/mcL
Basic metabolic panel with magnesium and phosphorus	
Sodium level	148 mmol/L high
Potassium level	4.1 mmol/L
Chloride	116 mmol/L high
Carbon dioxide	25 mmol/L
Glucose level	176 mg/dL high
Blood urea nitrogen	21 mg/dL
Creatinine	1.2 mg/dL
Calcium level	9.1 mg/dL
Magnesium level	2.3 mg/dL
Phosphorus level	1.1 mg/dL low

**Table 4:** Discharge lab results

Basic Metabolic Panel:	Hematology:
Sodium level: 145 mmol/L	Hemoglobin: 8.2 1/dL
Potassium level: 4.0 mmol/L	White blood cell count: 14.20 k/mcL
Phosphorus level: 2.9 mg/dL	Platelet count: 429 k/mcL
Magnesium level: 2.6 mg/dL	INR: 0.9
Blood urea nitrogen: 25 mg/dL	
Creatinine: 0.9 mg/dL	

## Discussion

The body employs a sophisticated internal mechanism to tightly regulate sodium levels that ensures equilibrium in serum osmolarity, blood pressure, and blood volume. Under normal physiologic conditions, an elevation of serum sodium levels will trigger an increase in ADH secretion and thirst sensation. Nevertheless, the inherent compensatory mechanisms of the body can sometimes fail, leading to hypernatremia. This is commonly caused by hypovolemia, resulting from free water loss either due to increased loss or reduced intake. However, when dealing with patients with hypopituitarism who lack adequate ability to secrete ADH, fluid replacement alone may not be sufficient to correct elevated sodium levels. On the contrary, some patients may have an inability to respond to ADH due to inadequate kidney function. As stated above, ADH, after being released from the posterior pituitary, travels to the kidneys, where adequate functioning of specific V2 receptors coupled to GPCR is needed to translocate aquaporin 2 to the renal apical membrane, increasing water reabsorption [14]. Thus, it is also important to group patients with chronic kidney disease into the discussion of consideration for monitoring sodium levels, as these patients too can rapidly deteriorate in acute hospital settings. In fact, according to a study done by Ranjan et al. in 2020, they found that patients with chronic kidney disease were far more likely to develop hypernatremia [15]. Thus, this case study may bring attention to a broader spectrum of patients and can be beneficial as a whole with an understanding of the ADH systemic mechanism of action and fluid shifts from volume depletion and repletion.

Upon admission, our patient also had a history of secondary adrenal insufficiency. The adrenal glands are vitally important for maintaining volume homeostasis, including important electrolytes such as sodium and potassium. This is done through the hormones aldosterone and cortisol. In short, aldosterone causes sodium to be reabsorbed and potassium to be excreted in the principal cells of the kidneys. Aldosterone's main stimulus, however, is the renin-angiotensin system, allowing our patient to still produce aldosterone [16]. Whereas cortisol directly suppresses ADH, causing ADH to be low during times of stress [17]. Our patient with hypopituitarism lacked ADH release in the first place and as such, the role of cortisol suppression was null. Regardless, one can see the vital interplay of two crucial hormones released from an important organ for the maintenance of electrolyte and fluid status, especially during times of stress such as infection from community-acquired pneumonia, as in our patient. All in all, it is clear that in hospital settings, particularly with elderly patients with multiple comorbidities, systemic function is not always aligned and functioning to its normal capacity. Thus, being able to adjust treatment based on a fundamental understanding of the hormonal interplay and chronic conditions was important in this patient and can be important in future patients with similar findings.

This case brings to light the importance of fully understanding the etiology of hypernatremic patients when treating them, as well as ensuring essential medications, such as DDAVP, are resumed in hypopituitarism patients when appropriate. Increasing awareness of the complications highlighted in this case could assist in the prevention of severe hypernatremia in future hypopituitarism patients presenting with CAP as well as other pathologies.

## Conclusions

This case presents a systematic approach to working up an acutely ill elderly patient with multiple comorbidities with life-threatening electrolyte abnormalities and infection. This case displays the uttermost importance of past medical history and how to treat a presenting illness step by step in order to eliminate the underlying issue. Oftentimes during hospital admissions, especially in comorbid patients, unpredictable and unexpected problems may occur and complicate the main presenting disease or illness. Understanding how to properly correct the issue is substantial and this case displays that with a very important electrolyte correction.

One must not only be able to identify the presenting signs and symptoms of hypernatremia but also understand its potentially fatal consequences, making it vital to act quickly and accordingly in the sense of proper correction. Using the appropriate calculated formula for water correction presented in this case as well as treating her reason for hospital admission saved this patient's life and should be considered when a similar problem presents itself, regardless of the underlying acute illness. As displayed in this case report, understanding physiological mechanisms with regard to volume balances, osmosis, and hormonal interplay all tie into the curative treatment of any patient.
